# A Baker’s Cyst With Distal Extramuscular Extension: A Case Report

**DOI:** 10.7759/cureus.77502

**Published:** 2025-01-15

**Authors:** Ervin P Anies, Robert A Waltz, Sean Lacey

**Affiliations:** 1 Physical Medicine and Rehabilitation, Walter Reed National Military Medical Center, Bethesda, USA; 2 Orthopedic Sports Medicine and Hip Arthroscopy, Naval Health Clinic Annapolis, Annapolis, USA; 3 Physical Medicine and Rehabilitation, Naval Health Clinic Annapolis, Annapolis, USA

**Keywords:** baker’s cyst, orthobiologics, popliteal cyst, prp, sclerotherapy, sports medicine, ultrasound

## Abstract

A 49-year-old man presented with repeated episodes of posterior knee effusions 11 months following right knee medial meniscus debridement. He was referred to Physical Medicine & Rehabilitation (PM&R) by his orthopaedic surgeon for further non-operative management. He had previously undergone ipsilateral vein stripping procedures with vascular surgery five months following his meniscal procedure. Magnetic resonance imaging (MRI) of the knee with contrast along with a subsequent ultrasound evaluation with no evident power Doppler flow confirmed the presence of a right posterior-medial gastrocnemius fluid collection extending extramuscularly into the proximal one-third of the medial head of the gastrocnemius muscle. These imaging findings combined with the yellow-clear aspirate consistent with synovial fluid confirmed the presence of a Baker’s cyst with unusually distal extramuscular extension and subcutaneous location just superficial to the medial head of the gastrocnemius muscle. Successful implementation of platelet-rich plasma (PRP) and doxycycline tissue sclerosis emphasized the key role that nonoperative modalities have in treating Baker’s cysts. The patient continues to respond appropriately with resolution of his symptoms noted three weeks after his initial sclerotherapy session and an additional treatment performed with half the initial dose of doxycycline. This case demonstrates the successful treatment of a Baker’s cyst with an unusually distal location.

## Introduction

A popliteal cyst, known as a “Baker’s cyst,” is a fluid accumulation in a capsular opening between the semimembranosus and the medial head of the gastrocnemius. Of note, these fluid collections are typically deep to the medial head of the gastrocnemius in the popliteal fossa. It is a common condition routinely discovered in 38% of magnetic resonance imaging studies (MRIs) performed on patients with symptomatic knees. Fluid can accumulate in this space secondary to intra-articular pathology such as osteoarthritis, chondral lesions, inflammatory arthropathy, and/or anterior cruciate ligament (ACL) tear [1].

Popliteal cysts are the most commonly diagnosed cystic structure within the knees of middle-aged and elderly populations [2]. Although clinical suspicion based on history, physical exam, and symptoms can be enough to suspect the diagnosis of a popliteal cyst, the gold standard for diagnosis is biopsy confirmation [2]. Despite this, non-invasive imaging in the form of MRI, computed tomography (CT) scan, and ultrasound (US) all remain as viable options in confirming the presence of a popliteal cyst [1-5]. However, even with the multitude of imaging modalities, MRI remains the standard [3,4]. In contrast to this standard, Neubauer et al. in 2011 and Singh et al. in 2021 proposed US as a considerable option in diagnosing these cysts [5,6]. The actual positioning of popliteal cysts depicted on MRI and US is elucidated in the discussion section below. 

Treatment options for popliteal cysts include US-guided aspiration and corticosteroid injection, with higher rates of recurrence noted in more complex cysts [2]. Although conservative management including corticosteroid injections has demonstrated effectiveness, some refractory cases go on to require surgery directed at the etiology of the effusion (e.g. meniscal pathology, chondral lesions) and closing or excision of the cyst itself [2].

## Case presentation

A 39-year-old male veteran with a right knee posterior medial meniscus debridement in 2021 and an ipsilateral right knee superficial vein stripping was referred to Physical Medicine & Rehabilitation (PM&R) due to right-sided posterior knee swelling, pain, and a noticeable fluid collection in the superficial to the mid-muscle belly of the medial head of the gastrocnemius. He had improved functionally until five months status-post his meniscal debridement. At five months, he still endorsed residual pain and right knee swelling with activities such as running and cycling, but these symptoms did not impede his ability to complete his activities of daily living (ADLs) both at work and at home. However, given the proximity in timing of his swelling to his meniscal surgery along with his superficial vein stripping surgery one month prior, the patient underwent a US with his vascular surgeon who diagnosed the mass as a seroma. 

At the time of referral to PM&R, the patient had completed his postoperative care and was following up with the attending orthopaedic surgeon when he described swelling and pressure in the back of his knee. On examination he was in no acute distress with no overlying skin changes noted over the palpable posterior right knee gastrocnemius fluid mass. His overall knee exam revealed structural integrity from a ligamentous, meniscal, and soft tissue examination. The patient had no restrictions with respect to range of motion and otherwise remained distally neurovascularly intact to all peripheral nerve distribution of his right lower leg. 

Scanning of the popliteal cyst

**Figure 1:**
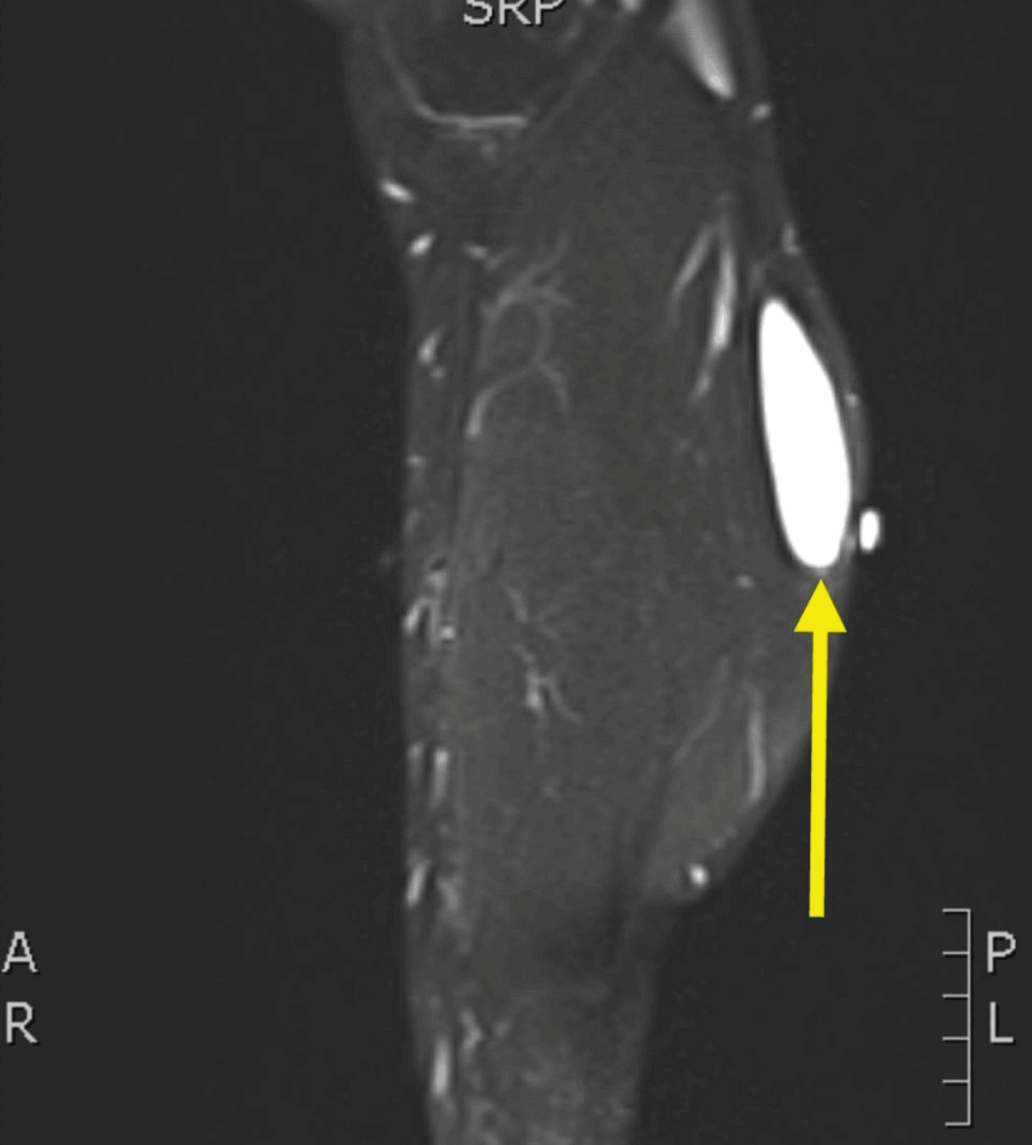
Non-contrast MRI sagittal (SAG) T2 fat suppressed (FS) image of the patient’s enhancing fluid collection demonstrating distal positioning and extramuscular extension.

**Figure 2:**
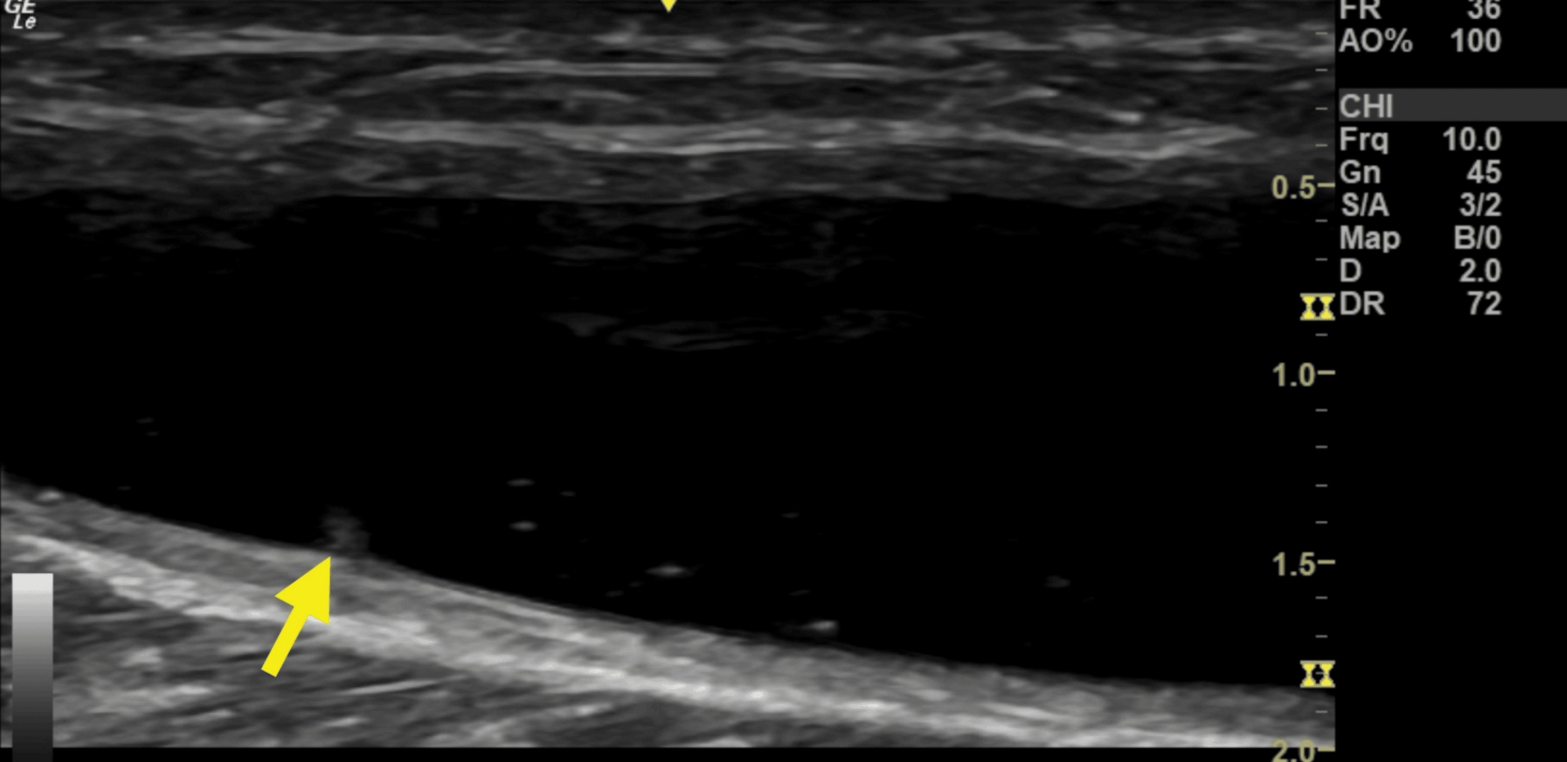
Sonographic image demonstrating sagittal, long axis view of anechoic, compressible fluid collection located 4-8 cm distal to the level of the popliteal fossa.

**Figure 3:**
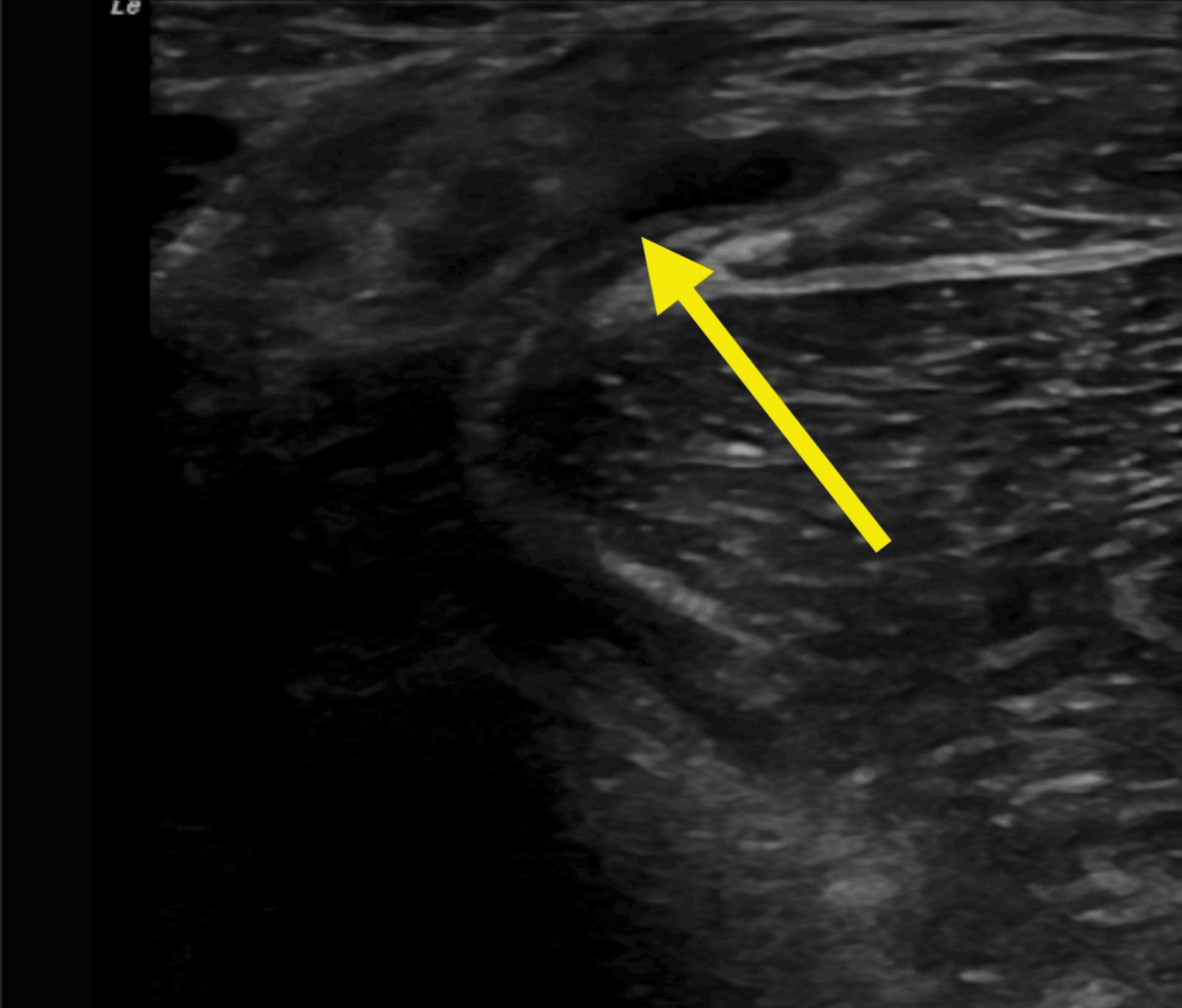
Sonographic image demonstrating axial, short axis view of the medial popliteal fossa region revealing an anechoic, compressible fluid collection between the medial head of the gastrocnemius muscle and semimembranosus, which was still visualized when adjusting for anisotrophy.

A previously performed non-contrast MRI of the right tib/fib was reviewed, which redemonstrated a multilobulated rim-enhancing fluid collection between the gastrocnemius and semimembranosus as well as a complex medial meniscus tear (Figure 1). Examination by the attending physiatrist was augmented by bedside point-of-care US, which revealed a hypoechoic, compressible fluid collection in the subcutaneous position which did not enhance with power Doppler evaluation (Figure 2). The fluid collection was scanned proximally where it was found to be continuous with the posterior knee joint in the expected position of a Baker’s cyst (Figure 3). 

Procedural technique

Following discussion between the physiatrist and orthopaedic surgeon, the patient underwent initial drainage of the cyst one year after his meniscus surgery, after the patient had dealt with his symptoms for seven months before undergoing treatment. A 25 gauge 1.5-inch needle was used to anaesthetize the skin with 1 mL of 2% lidocaine. A 21 gauge needle was advanced from proximal to distal under US guidance into the cyst at its most distal extent, being sure to avoid the medial sural cutaneous nerve. 23 mL of yellow, synovial fluid was aspirated without complication. The syringe was exchanged for the injectate of 2 mL 2% lidocaine to provide local pain relief for the patient. The patient was advised to wear ~18 mmHg thigh-high compression stockings as frequently as possible after the injection. 

At one-month follow-up, there was noted to be a reaccumulation of the fluid. He continued to deny any associated functional limitations but remained interested in both definitive surgical vs non-surgical treatment options given the symptom recurrence. Through a shared decision-making process, the patient elected to have a repeat aspiration with additional platelet-rich plasma (PRP) injection into the affected site as an inflammatory agent with the aim of adhering the cyst walls non-operatively. The procedure was scheduled to be completed two months after his initial cyst drainage. 

Subsequently, he opted to undergo PRP injection. As such, 60 mL of the patient's blood was placed in a centrifuge (Arthrex Angel, Naples, FL, USA) where it was spun down to 3 mL of leukocyte-rich PRP. A 21 gauge needle was advanced under US guidance into the cyst and with the use of one 60 mL syringe a total of 23 mL of yellow, synovial fluid was aspirated. The syringe was exchanged for the injection of 3 mL of PRP and the patient tolerated the procedure well without complication and was able to ambulate out of the clinic under his own power. 

At repeat follow-up appointment four weeks after the PRP injection, the patient’s cyst fluid had reaccumulated and the patient was counselled on pursuing doxycycline sclerosis as a treatment option for his cyst that had remained refractory. Despite continuing to have no functional limitations, the patient decided to elect for sclerosis which was scheduled to be completed one month after this appointment. The aim of this treatment was to provide relief of his proximal calf tightness which corresponded to the location of the cyst's most distal aspect.

In May 2023, the patient returned to the clinic where he again underwent review of his pertinent medical history, informed consent, and thorough discussion of the risks, benefits, and alternatives. In accordance with universal protocol, the patient was positioned comfortably in the prone position with knee extended. He was then prepped and draped in a sterile fashion, and a local skin wheal was raised with 1% lidocaine without epinephrine. There were no signs of infection at the injection site. Utilizing US guidance with a short axis view and in-plane approach, a 21 gauge needle was guided to the Baker's cyst at its extramuscular site in the posteromedial leg and was injected after negative aspiration of 30 mL of yellow fluid. The needle was maintained in place and replaced with 5 mL of sterile preservative-free normal saline reconstituted with 100 mg of doxycycline, which was injected into the cyst under direct US guidance. The fluid was spread throughout the cyst manually and the edges of the cyst were observed to be in tight approximation at the conclusion of the procedure. The patient tolerated the procedure well without any complications apart from the expected burning sensation post-procedurally. He was observed for 15 minutes throughout which time his burning sensation gradually improved. An ace wrap was used to tightly wrap the location and he was discharged from the clinic able to ambulate under his own power with post-procedure instructions.

Outcome and follow-up

At four months post doxycycline sclerosis, the patient was scheduled to follow up in clinic, but elected not to return to care for repeat evaluation due to satisfaction with the smaller size of the cyst. The only adverse event noted was significant burning at his injection site and around his knee, which resolved after 72 hours of rest. 

**Figure 4:**
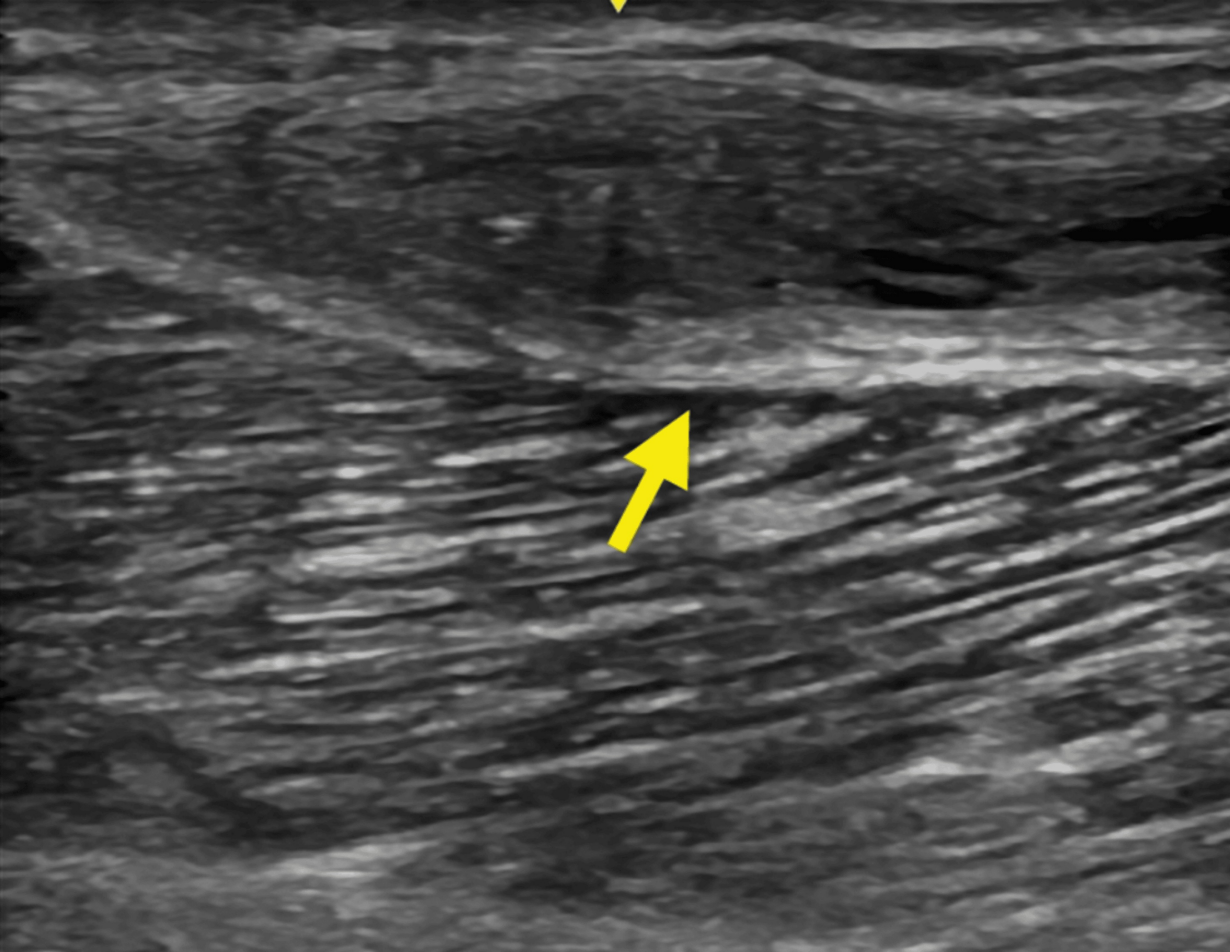
Sonographic image demonstrating sagittal / long axis view of the Baker's cyst status post doxycycline sclerosis demonstrating hyperechoic irregular synovial tissue within the cyst walls which was minimally compressible.

Two months later, the patient returned to the clinic requesting repeat clinical and sonographic evaluation and consideration of additional sclerotherapy treatment at a lower dose. The cyst was smaller in size, but he was interested in attempting a repeat treatment to further reduce the cosmetic appearance of the cyst (Figure 4). Repeat doxycycline sclerotherapy was performed with a further interval reduction in the proximal aspect of the cyst at follow-up six weeks later. At that time, he reported complete satisfaction with the outcome and no ongoing functional impairment related to the resolved cyst. We discussed that future treatment options would including surgical cystectomy vs. repeat doxycycline sclerotherapy.

## Discussion

On MRI, popliteal cysts are frequently visualized as ellipsoid masses with homogenous uniform low signal intensity on T1-weighted images as opposed to higher signal intensity on T2-weighted images [7,8]. They can either be unilocular or multilocular in nature and are commonly located between the semimembranosus tendon and medial gastrocnemius [9,10]. As typically depicted on axial MRI, there is a connection between the cyst itself and the subgastrocnemius bursa [7]. A connection is typically a pathophysiologic function of a primary injury (like the meniscal injury in our patient and the subsequent surgical debridement). The initial injury leads to a loss of structural integrity within the capsule thereby creating a secondary cystic structuring manifesting with a connection to the primary knee joint [7]. This is the basis for the one-way valvular mechanism as described by Rauschning et al. in 1978 [11]. This connection can be missing in certain diagnoses to include primary popliteal cysts (typically seen in the paediatric population) and in suspected malignancies or other benign solid tumours [7,12]. Although theoretically possible to extend in any direction, they most commonly extend inferomedially with it being much more rare to extend laterally or intramuscularly as demonstrated in case reports by Fang et al. and Kim et al. [9,10,13-16]. In contrast to the well-established literature on popliteal cysts and their typical positioning, our patient represents a uniquely documented popliteal cyst with distal extramuscular extension. 

In contrast to MRI, US typically depicts these cysts as anechoic or hypoechoic fluid visualized in the anatomic plane between the medial gastrocnemius and semimembranosus tendon with a recent meta-analysis suggesting accurate diagnosis even when transverse cyst diameters were less than 4 mm [1]. Being that this is commonly the presenting location for most cysts, Neubauer et al. described some other aspects include homogeneous hypoechoic internal signal, mixed echogenicity, variations in cyst wall thickenings, and occasional extraluminal fluid [16]. Given the predominance of popliteal cysts to extend within intermuscular planes inferomedially and rarely laterally or intramuscularly, cyst positionings diagnosed on US typically parallel those seen on MRI [15,16]. These mirrored imaging findings on both MRI and US help to accentuate the novelty of this patient’s case with associated extramuscular extension. 

Surgical treatment of Baker’s cysts can be considered if non-operative treatment fails over the course of several months, however has a historically high rate of recurrence and complications with cyst excision alone through either a limited or extended posterior medial approach [17,18]. One study reporting on 46 excisions found that 63% recurred and 33% suffered wound complications or pseudothrombophlebitis post-operatively [17,18]. Addressing intra-articular pathology through either meniscal debridement vs repair, cyst drainage through the capsular defect, and cyst drainage coupled with arthroscopic capsular wall excision have all been shown to be effective [17,19]. The most successful treatment is arthroscopic cyst wall excision through both standard and accessory posterior medial portals with 81.8% cyst absence on follow-up MRI [20] but also has the highest complication rate [19-21]. This underscores the importance of minimally invasive techniques such as the treatment described in this case to effectively treat Baker's cysts and minimize the complication risk to the patient. 

Doxycycline sclerotherapy has been used for ganglion cysts in the literature, with concern for chondrotoxicity and the potential for neurotoxicity if extravasated surrounding a nearby nerve [22,23]. However, there is no high-level data on doxycycline sclerotherapy for Baker's cysts specifically. 

Multidisciplinary collaboration between surgical and non-surgical specialists from a multitude of backgrounds assists in optimizing patient outcomes and ensuring the best possible return to function for patients. In this case, the surgical treatment offered by the orthopedist coupled with the vascular surgeon evaluation to rule out vascular etiology, augmented by physiatric evaluation to include bedside sonography, aspiration and ultimately sclerosis, led to the improved patient outcomes. 

## Conclusions

This case presents a popliteal cyst with extramuscular extension and unusual distal location, 8 cm below the popliteal fossa. The cyst’s position superficial to the gastrocnemius muscle in an unusual distal location is unique within the medical literature. Collaboration between physiatrists and orthopaedic surgeons was critical to properly evaluate the mass and treat it non-operatively with minimally invasive outpatient procedures. The diagnostic utility of point-of-care US is emphasized in the ability to characterize the symptomatic cyst and have live feedback for how to properly drain and sclerose the cyst itself. 

Nonoperative modalities such as corticosteroid injections and doxycycline-induced sclerosis of the lesion serve as viable treatment options for patients who do not wish to pursue surgical management or repeated drainages. This case helps to further add to popliteal cyst literature and establish a new perspective of how this common pathology can radiographically present extending distally in an extramuscular position as well.
